# Clinical relevance of the *cagA*, *tnpA* and *tnpB* genes in *Helicobacter pylori*

**DOI:** 10.1186/1471-230X-14-33

**Published:** 2014-02-19

**Authors:** Amin Talebi Bezmin Abadi, Ashraf Mohhabati Mobarez, Marc JM Bonten, Jaap A Wagenaar, Johannes G Kusters

**Affiliations:** 1 Department of Medical Microbiology, University Medical Center Utrecht, Heidelberglaan 100, Utrecht 3584 CX, The Netherlands; 2 Department of Medical Bacteriology, School of Medical Sciences, Tarbiat Modares University, Tehran, Iran; 3 Department of Infectious Diseases and Immunology, Faculty of Veterinary Medicine, Utrecht University, Utrecht, Netherlands

**Keywords:** *Helicobacter pylori*, Disease association, Gastric cancer, Duodenal ulcer, Virulence factor

## Abstract

**Background:**

Numerous proteins have been proposed as virulence factors for the gram negative gastric bacterium *Helicobacter pylori* but only for a few this has unequivocally been demonstrated. The aim of the current study was to evaluate the association of the putative virulence factors *tnpA* and *tnpB (no cagA)* with *H. pylori* associated gastroduodenal diseases.

**Methods:**

A PCR based assay was used to determine the presence of the *tnpA* and *tnpB* genes, as well as of *cagA*, in 360*H. pylori* strains isolated from *H. pylori* infected patients.

**Results:**

Of 360*H. pylori* culture positive patients (196 men, 164 women; average age 42.1 years (range 17–73), 95 had gastritis, 92 had gastric ulcers, 108 had duodenal ulcers, and 65 had gastric cancer. Using the gastritis group as a reference a significantly aberrant gene distribution was observed for the *tnpA* (Relative risk: 1.45; 95% CI 1.04-1.93)*,* the *cagA* (Relative risk: 1.81; 95% CI 1.44-2.29), but not the *tnpB* gene in the gastric cancer group.

**Conclusions:**

The increased incidence of the *tnpA* gene in gastric cancer patients suggests a role of the *tnpA* gene in the development of *H. pylori* induced gastric cancer.

## Background

*Helicobacter pylori* is the most prevalent pathogenic microorganism colonizing the gastric mucosa of humans. Infection rates range between 85-95% in developing countries and 30-50% in developed countries [1]. Colonization always results in acute gastritis and chronic gastritis when left untreated [2]. Additional complications such as gastric ulcers (GU), duodenal ulcers (DU), or gastric cancer (GC) may develop in some of these *H. pylori* infected patients [3]. The outcome of the infection is determined by both the duration of infection and environmental, host, and bacterial factors [4]. *H. pylori* strains display extensive genetic variability with considerable variation in the presence of virulence factors, which is thought to cause the many different clinical presentations of *H. pylori* infections [5-7]. The CagA protein is a commonly accepted virulence factor and the *cagA* gene is often used as a marker for the presence of the *cag* (cytotoxin-associated gene) pathogenicity island (*cag*PAI) [4]. Patients infected with *H. pylori* strains that carry *cagA* have a higher risk for developing peptic ulcer and gastric cancer [8]. Other virulence determinants located on the *cag*PAI such as *cagE, cagG, cagH, cagI, cagL*, and *cagM* are required for *cag*PAI mediated NF-κB induction, and *cagT* and *cagY* are required for the formation of a needle-like structure that serves to inject *cagA* into the host cell [9,10]. Although these factors play a critical role in the pathogenesis of *H. pylori,* their association with specific disease outcomes is not as obvious as with *cagA*. It has been reported that in some *H. pylori* strains the *cag*PAI is split into two separate regions due to the integration of the IS*605* insertion sequence [10]. The putative IS*605* transposases (*tnpA* and *tnpB*) that can mediate this *cag*PAI disruption [10] might affect the virulence of *H. pylori*[11], but the exact biological role and clinical relevance of these two determinants is poorly studied. Iran is a developing country with a high prevalence of *H. pylori,* among both symptomatic and asymptomatic individuals, and with a prevalence as high as 95% in the northern part of the country [12,13]. This high prevalence is coupled to an even higher rate of *H. pylori* induced peptic ulcer disease and gastric cancers [14]. This makes it an ideal geographically confined region to study the effect of genetic variation of this gastric pathogen on infection associated disorders. In this study we determined associations of the presence of *tnpA* and *tnpB* and clinical manifestations of *H. pylori* infections in patients from the North of Iran.

## Methods

### Patients

All patients suspect of a *H. pylori* infection that visited the Tooba Medical Center, in Sari, Iran for endoscopic examination between May 2008 and October 2010 were invited to participate in this study. Patients participating in this study underwent routine gastroscopic examination. The standard number of gastric biopsy samples for patients’ suspect of Helicobacter infection was obtained for routine culture and histological investigations and no extra samples were taken for this study. One of the gastric biopsy samples was sent to the pathology lab where it was tested by routine histopathological techniques and evaluated by standard criteria. Histology grading was performed by the updated Sydney criteria [15]. The other routinely obtained biopsy samples were used for microbiological culture and Rapid Urease Test (RUT), as described below. Ages below 16 years were excluded due to ethical considerations. Also antibiotic use within four months prior to endoscopy, or use of anti-secretory drugs within one month before endoscopy were used as exclusion criteria. This study was approved by the local ethics committee of Tarbiat Modares University, as no extra biopsy samples were needed for this study and that the obtained data could not be traced back to the patient level.

### Microbiological analysis

One of the biopsy samples was routinely tested by the gastroenterologist by Rapid Urease Test; and if positive a second sample was obtained and placed in 200 μl sterile thioglycolate (Merck, Germany) broth and then immediately shipped to the diagnostic laboratory for routine culture. Upon arrival in the microbiology lab this sample was immediately grinded and 100 μl of the resultant homogenate was inoculated on a Colombia agar (Merck, Germany) plate supplemented with 7% defibrinated sheep blood (Jihad Daneshgahi, Tehran, Iran), 10% Fetal Calf Serum (FCS) and antibiotics (DENT, Supplement, Oxoid) [15]. Plates were incubated at 37°C, in 10% CO_2_ conditions provided by incubator (Binder, USA) and high humidity until typical *H. pylori* colonies appeared or for a maximum of 7 days if no suspect colonies were observed. Colony shape, morphology in microscopic examination, routine biochemical tests such as urease, catalase and oxidase tests were performed for identification of *H. pylori* strains.

### DNA extraction and PCR

Bacterial DNA was extracted from single colonies of *H. pylori* using a commercially available kit (ExiPrep™ Bacteria Genomic DNA Kit, Bioneer, Daejeon, South Korea). Genotyping was performed by PCR, using specific primers for *cagA, tnpA* and *tnpB* as previously described (Table 1). In addition a *glmM* PCR (Table 1) was carried out [16], both as an additional control for *H. pylori* identification and quality check of the isolated DNA (positive PCR control). The PCR amplified fragments were size separated on 2% agarose gel (Sinagene, Tehran, Iran) and the ethidium bromide stained DNA was visualized using UV illumination.

**Table 1:** Primers used in this study

**Primers**	**5′-3′ Sequence**	**Reference**
***glmM***	AAGCTTTTAGGGGTGTTAGGGGTTT	[20]
	AAGCTTACTTTCTAACACTAACGC	
***tnpA***	ATCAGTCCAAAAAGTTTTTTCTTTCC	[13]
	TAAGGGGGTATATTTCAACCAACCG	
***tnpB***	CGCTCTCCCTAAATTCAAAGAGGGC	[13]
	AGCTAGGGAAAAATCTGTCTATGCC	
***cagA***	ATAATGCTAAATTAGACAACTTGAGCGA	[5]
	TTAGAATAATCAACAAACATCACGCCAT	

### Statistical analysis

The chi-square and Fisher exact test was used to test for the association between patient demographics, *H. pylori* genotypes, and disease groups. A *P* value of less than 0.05 was accepted as statistically significant. Microsoft Excel 2010 was used to calculate the *P* values, odds ratio (OR) and 95% confidence interval (95% CI).

## Results

376 patients suspect for *H. pylori* infection (positive RUT test) were enrolled, but *H. pylori* specific growth was not observed from the biopsy specimen in 16 of them. The remaining 360 patients that were *H. pylori* culture positive (96%) comprised 95 patients with gastritis (G), 92 with gastric ulcer (GU), 108 with duodenal ulcer, and 65 with gastric cancer (GC) (Table 2). The average age was 42.1 years (range 17 to 73 year) and there were slightly more men (n = 196) than women (n = 164). Detailed demographic data of dyspeptic patients according to age, disease symptoms, and histological findings are shown in Table 2. There were slightly more males with duodenal ulcers, and less with gastric ulcers, but there were no statistically significant associations between age, gender, histopathological findings, and *H. pylori* associated disease groups*.*

**Table 2:** Detailed demographic data of dyspeptic patients according to the age and pathologic findings

**Disease type**	**Sample size**	**Male (%)**	**Pathology findings**	**Age range detailed data for each disease groups**				
				**<30**	**31-40**	**41-50**	**51-60**	**>60**
**G**	**95**	**51 (53.6)**	Mild (n = 14)	6	7	1	0	0
			Moderate (n = 67)	33	26	6	2	0
			Atrophic (n = 20)	8	5	6	1	0
**GU**	**92**	**38 (41.3)**	Mild (n = 15)	2	3	4	4	2
			Moderate (n = 84)	7	12	21	11	33
			Atrophic (n = 13)	0	2	2	6	3
**DU**	**108**	**72 (66)**	Mild (n = 23)	4	5	5	6	3
			Moderate (n = 57)	6	18	13	12	8
			Atrophic (n = 23)	6	7	7	6	4
**GC**	**65**	**35 (53.8)**	Mild (n = 7)	0	0	1	2	4
			Moderate (n = 47)	0	0	17	16	14
			Atrophic (n = 11)	0	0	6	1	4

### PCR screening of *tnpA, tnpB* and *cagA*

The overall prevalence of the *tnpA, tnpB,* and *cagA* genes were 47.5%, 13.1%, and 59.2%, respectively, and the prevalence of these genes in the four disease groups is listed in Table 3. No significant associations were observed between the presence of the *tnpA, tnpB* and *cagA* genes and histological findings. Statistical analysis did however reveal a significant association between the presence of the *cagA* gene and GC [Relative risk: 1.81; 95% CI 1.44-2.29], and a weak, but significant correlation was observed between the presence of the *cagA* gene and DU [Relative risk: 1.30; 95% CI 1.01-1.69] and the *tnpA* gene with GC [Relative risk: 1.45; 95% CI 1.04-1.93] (Table 3). No significant association was observed for *tnpB* and gastroduodenal diseases.

**Table 3:** **Prevalence of the *tnpA*, *tnpB*, and *cagA *** **genes in the four patient groups**

**Disease groups**	***tnpA***			***tnpB***			***cagA***		
	**Positives**	**Relative risk**	**95% CI**	**Positives**	**Relative risk**	**95% CI**	**Positives**	**Relative risk**	**95% CI**
Gastritis (n = 95) (Control group)	40 (42.1%)	Reference		16 (16.8%)	Reference		45 (47.4%)	Reference	
Gastric ulcer (n = 92)	48 (52.2%)	1.23	0.97-1.61	10 (10.9%)	0.64	0.30-1.34	45 (48.9%)	1.03	0.76-1.39
Duodenal ulcer (n = 108)	44 (40.7%)	0.96	0.69-1.34	11 (10.2%)	0.59	0.29-1.22	67 (62%)	1.30	1.01-1.69
Gastric cancer (n = 65)	39 (60.0%)	1.45	1.04-1.93	10 (15.4%)	0.91	0.44-1.88	56 (86.2%)	1.81	1.44-2.29

## Discussion and conclusions

To our knowledge, this is the largest study (n = 360) investigating the distribution of the *H. pylori* virulence *tnpA, tnpB* and *cagA* in dyspeptic patients. In the first study on *tnpA* and *tnpB* by Matter *et al.*[11], 63% of 215 clinical *H. pylori* isolates were *tnpA* positive and 13.5% were positive for *tnpB*, with a statistically significant association between peptic ulcer disease (PUD) and *tnpA* positive strains. This association was not apparent for *tnpB*. In the current study there was a similar prevalence of *tnpA* and *tnpB* [171/360; 47.5% and 47/360; 13.1%, for *tnpA* and *tnpB* respectively], and a similar association between *cagA* and gastric cancer patients as observed in a preliminary study by Matter *et al.*[11]. Unfortunately in their study the associations of *tnpA* and *tnpB* with *H. pylori* associated disease types were not determined. In a more recent but smaller study Matter *et al.* investigated associations between presence of *tnpA* and *tnpB* and gastric cancer in Brazilian patients with gastric cancer (n = 34) and gastritis (n = 34) [17]. The patient population studied here is from the North of Iran (state of Mazandaran), and has been reported to not only have a very high *H. pylori* infection rate but also a very high rate patients suffering from *H. pylori* induced disease [18] This high *H. pylori* prevalence facilitates the collection of a large number of strains from a well defined, small geographical region and this facilitated a study on the putative association between the presence of tnpA/B and the clinical outcome of the *H.pylori* infection. The prevalence of *tnpA* and *tnpB* among gastric cancer and gastritis patients in the Iranian population included in the current study was 42.1% and 60.0% for *tnpA*, and 16.8% and 15.4% for *tnpB,* respectively, which, again, was comparable to the findings in the Brazilian population with gastric cancer or gastritis (29.4% and 73.5% for *tnpA*; and 2.9% and 5.9% for *tnpB,* respectively). Kersulyte *et al.*[19] also reported a higher frequency of *tnpA* in Peruvian gastric cancer strains than in gastritis strains (9/14 (46%) versus 15/45 (33%), respectively). Although the observed associations between *tnpA* and gastric cancer are similar in the populations in Peru, Brazil and Iran [11,19], there are striking differences for associations of *cagA* with disease status between these populations. We observed a clear association between the presence of *cagA* and gastric cancer in the Iranian population, while Matter *et al.*[11] did not observe such an association in Brazil. While most studies report an association between the presence of *cagA* and gastric cancer some studies do not observe this association [20,21]. In this particular case it may be due to the low number of patients included in their study (n = 64; versus 160 in our study). After the recognition of *H. pylori* as an important gastric pathogen [21], many attempts have been made to identify *H. pylori* virulence factors predicting clinical outcome as this might assist physicians in prediction of disease progression [22]. When using the gastritis group as controls for gene distribution we observed an increased prevalence of the *tnpA* and *cagA* genes in the gastric cancer group. To our knowledge this study represents the largest cohort tested thus far for the prevalence of *tnpA* for an association with the various *H. pylori* infection associated disease groups. While it is tempting to conclude from the increased prevalence of *tnpA* and *cagA* in the gastric cancer group that these genes may serve as useful biomarkers for gastric cancer one cannot draw that conclusion from a cross-sectional study like ours. Also our study design did not include a questionnaire on the disease history of our patients and hence we are unable to correlate clinical symptoms such as bleeding, reflux, abdominal pain etc. with the presence/absence of these virulence factors. A large prospective cohort study would be required to establish reliable positive and negative predictive values of these putative biomarkers. Due to the long time between infection and cancer development such a study would require long follow-up times, and since only few infected individuals develop cancer a large study cohort would be required. In addition there are ethical issues with such a study as the hypothesis to be tested is that patients infected with *tnpA* positive *H. pylori* strains are more prone to developing gastric cancer than patients infected with *tnpA* negative strains. In order to test this hypothesis one must establish the presence of the *tnpA*^*+/−*^*H. pylori* strain at the start of the study while refraining from eradication of these potentially carcinogenic strains for a long period of time. In spite of the shortcomings of our cross-sectional study it provides strong indications for the clinical relevance of the *tnpA* gene of *H. pylori* strains isolated from the Iranian population where the prevalence of *H. pylori* is relatively high [13] and this high prevalence is coupled to a high incidence of *H. pylori* induced peptic ulcer disease and gastric cancers [23]. In conclusion *tnpA* but not *tnpB* is clearly associated with a more severe disease outcome of *Hc pylori* infections. As such *tnpA* could be a valuable novel biomarker but clearly further studies are required to confirm these results especially since at present no obvious biological explanation for a GC inducing function of this putative transposase can be provided.

## Competing interests

The authors declare that they have no competing interests.

## Authors’ contributions

ATBA, JGK and AMM build the strain collection, collected the patient status data, carried out the molecular genetic studies, performed the initial statistical analyses, and drafted the manuscript. ATBA, JGK, JAW and MJB participated in the design of the study, the drafting of the mansuscirpt and assisted with the statistical analysis. All authors read and approved the final manuscript.

## Pre-publication history

The pre-publication history for this paper can be accessed here:

http://www.biomedcentral.com/1471-230X/14/33/prepub
